# Understanding Breast-Cancer-Associated Fibroblasts and Their Epigenetic Activation to Unveil Novel Targets for Breast Cancer Therapy

**DOI:** 10.3390/cancers15164073

**Published:** 2023-08-12

**Authors:** Aamir Ahmad

**Affiliations:** 1 Translational Research Institute, Academic Health System, Hamad Medical Corporation, Doha 3050, Qatar; aamirahmad100@gmail.com; Tel.: +974-44390984; 2 Dermatology Institute, Academic Health System, Hamad Medical Corporation, Doha 3050, Qatar; 3 Department of Dermatology and Venereology, Rumailah Hospital, Hamad Medical Corporation, Doha 3050, Qatar

The review article ‘Cancer-Associated Fibroblasts: Epigenetic Regulation and Therapeutic Intervention in Breast Cancer’ by Lee et al. [1] discusses the evolving potential of cancer-associated fibroblasts (CAFs) as potential therapeutic targets, particularly in breast cancer. The focus on breast cancer is understandable considering the incidence as well as mortality associated with breast cancer. As per the 2023 statistics in the United States [2], breast cancer is expected to lead all cancers among women in terms of incidence, accounting for almost one-third of all new cancer diagnoses. While this information is scary, a positive note is that even though the incidence rate has been increasing for the past several years, mainly due to more aggressive screenings, the mortality rate has not increased at the same pace [3], suggesting an improvement in the overall management of the disease. However, the number of breast-cancer-associated deaths are still too high, both in the US and worldwide, and more robust targets for therapy need to be identified and characterized.

Among the many aspects of a growing tumor, the tumor microenvironment (TME) has gained a lot of interest recently because it represents a close and protective niche of many different cell types that protect tumor cells from therapies [4] and support their growth, proliferation and invasion. The molecular biomarkers of focus in the article by Lee et al. [1] are fibroblasts, specifically CAFs, which represent a major component on TME. CAFs are specialized, activated fibroblasts that support tumor growth; they have considerable heterogeneity and plasticity [5]. Though it is widely believed that CAFs originate from ‘normal’ tissue-resident fibroblasts, there are examples of alternate origins of CAFs as well [6]. CAFs can be identified based on their spindle-shaped morphology. However, this morphology can be confused with the cancer cells undergoing epithelial-mesenchymal transition (EMT); the lack of endothelial, epithelial, and leukocyte markers’ expressions, in addition to the absence of specific cancer mutations, helps to identify CAFs [1,6]. One way that CAFs support tumor growth is through the release of growth factors, cytokines and chemokines [7,8,9]. It has been estimated that up to 70% of a breast tumor could be just CAFs, or more specifically, ‘breast cancer-associated fibroblasts (BCAFs)’ [1,10]. The crosstalk between BCAFs and breast tumor cells is bi-directional. As discussed, BCAFs secrete multiple factors to aid tumor progression. On their part, tumor cells release factors such as platelet-derived growth factors (PDGFs) and transforming growth factor-beta (TGF-β), which help the activation of BCAFs [11].

The article by Lee et al. [1] discusses in detail the heterogeneity of BCAFs because this is one area of research on CAFs that has attracted a lot of attention. The heterogeneity is apparent in the diverse functionality of different BCAF subtypes. Interestingly, there seems to be a direct effect of BCAF–breast-cancer cells’ bidirectional crosstalk on CAF heterogeneity as well, as supported by the observations that breast cancer with different mutations can activate BCAFs differentially, giving rise to BCAFs with diverse functionalities [12]. As an example, BCAFs activated by aggressive breast tumors with high migration capability can make breast cancer cells with relatively lower migration ability more invasive [1,12]. The article [1] discusses the different classifications and subtypes of CAFs. It discusses pro- vs. anti-tumorigenic CAFs. It also discusses four BCAF subtypes depending on the differential expression of specific biomarkers [13] and four BCAF subtypes depending on the different precursor cells that activated the BCAFs initially [14]. The article [1] also provides an overview of genetic changes that are apparent in the activation of CAFs from their precursor normal fibroblasts. 

After the initial discussions on CAFs’ origin, heterogeneity, etc., the discussion moves to the epigenetic regulation of BCAFs. It is mentioned that the epigenetic regulation of normal fibroblasts leading to activated fibroblasts can be reversible or irreversible. A reversible activation leads to normal activated fibroblasts, whereas CAFs are the result of irreversible activation [1,15]. It is also pointed out that exosomes may play a role in the epigenetic activation of BCAFs through shuttling factors that can bring about epigenetic changes [16]. Among the specific epigenetic events, the changes in DNA methylation and the resulting impact are discussed. For example, differential promoter methylation can impact gene expressions and contribute to tumors supporting and the acquisition of pro-tumorigenic phenotypes of CAFs [17]. Specific examples of several genes are provided; additionally, the emerging concept that the available literature has primarily focused on over-expressed genes in activated CAFs because of their hypomethylation in CAFs, or conversely their hypermethylation in normal fibroblasts, is discussed. The observations might be biased towards oncogenic genes that are overexpressed in CAFs, as compared to their precursors. The article by Lee et al. [1] also discusses the post-translational modifications of histone protein in BCAFs. These modifications represent another major epigenetic event [18] with evidence of differential histone methylation leading to the secretion of A disintegrin and metalloproteinase with thrombospondin motifs 1 (ADAMTS1) and the inhibition of the repressor Enhancer of zeste homolog 2 (EZH2) [19]. Finally, the discussion on the epigenetic regulation of BCAFs moves to microRNAs (miRNAs). As pointed out in the article, this regulation represents one of the most well-studied epigenetic regulations of BCAFs. A few miRNAs are identified in the discussion and, among them, the miR-200 family of miRNAs stands out as a prominent one. miR-200 is a tumor suppressor the miRNA family that negatively correlates with EMT and breast cancer aggressiveness [20]. As expected, this miRNA family is downregulated in BCAFs [21]. The authors summarize a number of miRNAs in a tabular form. It is concluded that while some miRNAs, such as, miR-31, miR-221, miR-9, miR-21, miR-155, miR-143 and miR-378e, are upregulated in BCAFs, other miRNAs, such as, miR-200 family miRNAs, miR-320, let-7g, miR-31, miR-26b and miR-148a, are downregulated in BCAFs [1].

With the identification of mechanisms that lead to the activation of BCAFs, it makes sense to exploit the information to validate novel targets for therapy. This is covered by Lee et al. [1] in the last section of their article. They point out the clinicals trials evaluating epigenetic drugs such as DNA methyltransferases inhibitors (DNMTi) and histone deacetylases inhibitors (HDACi) [22]. Several DNMTis and HDACis are discussed, with the eventual recognition that some of these inhibitors have been tested in phase 1 and phase 2 clinical trials [1,23]. Further, even though most of the in vitro work on the epigenetic regulation of BCAFs has focused on miRNAs, there is a clear lack of evaluation of miRNAs as targets of therapy in clinics; this is primarily due to the lack of miRNA-targeted therapies that can be tested in clinics in patients with breast cancer. The factors released by BCFAs within the TME also present as attractive targets of therapy. Additionally, a better understanding of tumor-suppressive CAF phenotypes can potentially help identify novel targets for therapy. 

In summary, the article by Lee et al. presents a comprehensive overview of CAFs, specifically BCAFs, with discussion on BCAFs’ origins/heterogeneity, bi-directional communications with breast tumor cells, their epigenetic regulation and the identification of key factors in their activation that have the potential to be further developed into valid targets for therapy. 

## References

[1] Lee Y.T., Tan Y.J., Falasca M., Oon C.E. (2020). Cancer-Associated Fibroblasts: Epigenetic Regulation and Therapeutic Intervention in Breast Cancer. Cancers.

[2] Siegel R.L., Miller K.D., Wagle N.S., Jemal A. (2023). Cancer statistics, 2023. CA Cancer J. Clin..

[3] Ahmad A. (2019). Breast Cancer Statistics: Recent Trends. Adv. Exp. Med. Biol..

[4] Ahmad A. (2022). Tumor microenvironment and immune surveillance. Microenviron. Microecol. Res..

[5] Ping Q., Yan R., Cheng X., Wang W., Zhong Y., Hou Z., Shi Y., Wang C., Li R. (2021). Cancer-associated fibroblasts: Overview, progress, challenges, and directions. Cancer Gene Ther..

[6] Sahai E., Astsaturov I., Cukierman E., DeNardo D.G., Egeblad M., Evans R.M., Fearon D., Greten F.R., Hingorani S.R., Hunter T. (2020). A framework for advancing our understanding of cancer-associated fibroblasts. Nat. Rev. Cancer.

[7] Gascard P., Tlsty T.D. (2016). Carcinoma-associated fibroblasts: Orchestrating the composition of malignancy. Genes Dev..

[8] Hassan M.S., Cwidak N., Awasthi N., von Holzen U. (2022). Cytokine Interaction With Cancer-Associated Fibroblasts in Esophageal Cancer. Cancer Control.

[9] Mao X., Xu J., Wang W., Liang C., Hua J., Liu J., Zhang B., Meng Q., Yu X., Shi S. (2021). Crosstalk between cancer-associated fibroblasts and immune cells in the tumor microenvironment: New findings and future perspectives. Mol. Cancer.

[10] Louault K., Bonneaud T.L., Seveno C., Gomez-Bougie P., Nguyen F., Gautier F., Bourgeois N., Loussouarn D., Kerdraon O., Barille-Nion S. (2019). Interactions between cancer-associated fibroblasts and tumor cells promote MCL-1 dependency in estrogen receptor-positive breast cancers. Oncogene.

[11] Ostman A. (2014). Cancer-associated fibroblasts: Recent developments and emerging challenges. Semin. Cancer Biol..

[12] Tchou J., Kossenkov A.V., Chang L., Satija C., Herlyn M., Showe L.C., Pure E. (2012). Human breast cancer associated fibroblasts exhibit subtype specific gene expression profiles. BMC Med. Genom..

[13] Costa A., Kieffer Y., Scholer-Dahirel A., Pelon F., Bourachot B., Cardon M., Sirven P., Magagna I., Fuhrmann L., Bernard C. (2018). Fibroblast Heterogeneity and Immunosuppressive Environment in Human Breast Cancer. Cancer Cell.

[14] Bartoschek M., Oskolkov N., Bocci M., Lovrot J., Larsson C., Sommarin M., Madsen C.D., Lindgren D., Pekar G., Karlsson G. (2018). Spatially and functionally distinct subclasses of breast cancer-associated fibroblasts revealed by single cell RNA sequencing. Nat. Commun..

[15] Kalluri R. (2016). The biology and function of fibroblasts in cancer. Nat. Rev. Cancer.

[16] Baroni S., Romero-Cordoba S., Plantamura I., Dugo M., D’Ippolito E., Cataldo A., Cosentino G., Angeloni V., Rossini A., Daidone M.G. (2016). Exosome-mediated delivery of miR-9 induces cancer-associated fibroblast-like properties in human breast fibroblasts. Cell Death Dis..

[17] Sylvestre M., Tarte K., Roulois D. (2020). Epigenetic mechanisms driving tumor supportive microenvironment differentiation and function: A role in cancer therapy?. Epigenomics.

[18] Zhao Z., Shilatifard A. (2019). Epigenetic modifications of histones in cancer. Genome Biol..

[19] Tyan S.W., Hsu C.H., Peng K.L., Chen C.C., Kuo W.H., Lee E.Y., Shew J.Y., Chang K.J., Juan L.J., Lee W.H. (2012). Breast cancer cells induce stromal fibroblasts to secrete ADAMTS1 for cancer invasion through an epigenetic change. PLoS ONE.

[20] Ahmad A., Aboukameel A., Kong D., Wang Z., Sethi S., Chen W., Sarkar F.H., Raz A. (2011). Phosphoglucose isomerase/autocrine motility factor mediates epithelial-mesenchymal transition regulated by miR-200 in breast cancer cells. Cancer Res..

[21] Tang X., Hou Y., Yang G., Wang X., Tang S., Du Y.E., Yang L., Yu T., Zhang H., Zhou M. (2016). Stromal miR-200s contribute to breast cancer cell invasion through CAF activation and ECM remodeling. Cell Death Differ..

[22] Stahl M., Kohrman N., Gore S.D., Kim T.K., Zeidan A.M., Prebet T. (2016). Epigenetics in Cancer: A Hematological Perspective. PLoS Genet..

[23] Munster P.N., Thurn K.T., Thomas S., Raha P., Lacevic M., Miller A., Melisko M., Ismail-Khan R., Rugo H., Moasser M. (2011). A phase II study of the histone deacetylase inhibitor vorinostat combined with tamoxifen for the treatment of patients with hormone therapy-resistant breast cancer. Br. J. Cancer.

